# Predictors of multidrug-resistant *Acinetobacter baumannii *infections: a retrospective analysis in surgical ICU patients

**DOI:** 10.1186/cc10651

**Published:** 2012-03-20

**Authors:** A Camkiran, A Kundakci, C Araz, A Pirat, P Zeyneloglu, H Arslan, G Arslan

**Affiliations:** 1 Baskent University, Ankara, Turkey

## Introduction

Multidrug-resistant *Acinetobacter baumannii *(MRAB) is an important cause of hospital-acquired infection and leads to an increasing morbidity and mortality in ICUs. The aim of this study was to investigate the predictors of MRAB infection in surgical ICU patients.

## Methods

The charts of the patients who were admitted to the ICU between January 2008 and August 2010 were reviewed to identify patients with MRAB infection. Recorded data were as follows: age, sex, medical history, underlying surgical pathology, APACHE II score on ICU admission, days in hospital before ICU, presence of invasive procedures (intubation, tracheostomy, arterial, central venous lines, urinary and nasogastric catheters, enteral or parenteral nutrition and renal replacement therapy), days in the ICU and white blood cell (WBC) count on infection day, infection site, complications (such as organ/system failure), length of stay (LOS) in the ICU and hospital, and final outcome.

## Results

During the study period 25 patients with MRAB infection were identified. When compared with their matched control group (*n *= 25), patients with MRAB infection had a significantly higher mean APACHE II score (*P *< 0.001) and more frequently had an open wound (*P *= 0.002) or required mechanical ventilation (*P *= 0.005), arterial catheterization (*P *= 0.006), and central venous catheterization (*P *= 0.004). Multivariate logistic regression revealed that APACHE II score (OR, 1.19; 95% CI, 1.005 to 1.315; *P *= 0.043) and open wound (OR, 0.45; 95% CI, 0.003 to 0.587; *P *= 0.18) were predictors of MRAB infection in these patients. Compared to their controls, patients with MRAB infection had a longer LOS in the ICU (36.44 ± 30.44 days vs. 7.80 ± 8.13 days, *P *< 0.001) and hospital (55.12 ± 40.81 days vs. 19.04 ± 13.44 days, *P *< 0.001). In-hospital mortality rates for patients with MRAB infection and their controls were 56% and 32%, respectively (*P *= 0.154).

## Conclusion

Our results indicate that higher APACHE II scores and presence of an open wound are predictors of MRAB in ICU surgical patients. Patients with MRAB infection tended to have a higher mortality and had a longer LOS in the ICU and hospital than their controls.

